# With a little help from my friends? Acculturation and mental health in Arabic-speaking refugee youth living with their families

**DOI:** 10.3389/fpsyt.2023.1130199

**Published:** 2023-03-15

**Authors:** Caroline Meyer, Lina Alhaddad, Nadine Stammel, Frederick Sixtus, Jenny Sarah Wesche, Rudolf Kerschreiter, Patricia Kanngiesser, Christine Knaevelsrud

**Affiliations:** ^1^Faculty of Education and Psychology, Freie Universität Berlin, Berlin, Germany; ^2^Department of Educational Psychology, Martin Luther University of Halle-Wittenberg, Halle (Saale), Germany; ^3^Berlin Institute for Population and Development, Berlin, Germany; ^4^School of Psychology, University of Plymouth, Plymouth, United Kingdom

**Keywords:** depression, posttraumatic stress, PTSD, adolescents, minors, Syria, social support, language

## Abstract

**Introduction:**

Refugee youth are often faced with the compounding challenges of heightened exposure to traumatic events and acculturating to a new country during a developmental period when their sense of self is still forming. This study investigated whether refugee youth’s acculturation orientation (separation, integration, marginalization, and assimilation) is associated with depressive and posttraumatic stress symptoms and aimed to identify additional indicators of acculturation that may contribute to mental health.

**Methods:**

A total of 101 Arabic-speaking refugee youths (aged 14–20 years), who were living with their families and attending school in Germany, took part in the study. They answered questions concerning traumatic exposure and posttraumatic stress symptoms, depressive symptoms, and several indicators of acculturation, including cultural orientation, positive and negative intra- and intergroup contact, language skills and friendship networks. All participants were categorized into one of four acculturation orientations using median splits.

**Results:**

Kruskal–Wallis rank sum tests revealed that acculturation orientation was not significantly associated with depressive symptoms [χ^2^ (3, 97) = 0.519, *p* = 0.915] or posttraumatic stress symptoms [χ^2^ (3, 97) = 0.263, *p* = 0.967]. Regression analysis revealed that German language skills were significantly associated with lower scores of depressive symptoms (*p* = 0.016) and number of friends in Germany was significantly associated with lower scores of depressive (*p* = 0.006) and posttraumatic stress symptoms (*p* = 0.002), respectively.

**Discussion:**

Policies that provide refugee youth with access to language classes and social activities with peers do not only enable them to actively participate in a new society but may also have a positive effect on their mental health.

## 1. Introduction

In recent years, the number of people who have been forced to migrate has increased dramatically worldwide. At the end of 2021, the United Nations High Commissioner for Refugees estimated that approximately 89.3 million people had been forcibly displaced from their homes and that 41% of them were minors (1). Refugee youth, compared to non-refugee youth, show increased rates of short- and long-term mental health problems such as post-traumatic stress disorder (PTSD), depression, or anxiety disorders (2, 3). Moreover, resettlement in a new country can be particularly challenging for youth as, in addition to acculturative challenges, they also experience developmental changes such as puberty, renegotiating relationships with their parents, and forming a sense of self (4–6).

Most research on refugee youth mental health to date has focused on unaccompanied youth, who face a particularly high risk of experiencing adverse events and trauma during flight and must face post-migration stressors without their primary caregivers (7–9). This often results in higher rates of mental health problems (10, 11). However, refugee youth living with their families also show elevated levels of mental health problems (9, 12) and share several risk factors with unaccompanied youth such as traumatic exposure in their home country and during their flight, and/or being separated from relatives (13, 14).

Refugee youth living with their parents are also exposed to other potential stressors. Due to the challenges experienced during their flight, refugee parents may experience elevated levels of mental health problems (15), which in turn have been associated with mental health problems in children and youth (16, 17). Refugee youth living with their families often have to navigate two worlds in their everyday life, one at home and one outside their home. While this allows them to develop unique skills, such as flexibility, adaptability, and empathy (18), it can be challenging when socio-cultural expectations vary drastically between these two “worlds” and when refugee youth and their parents adjust differently to life in the resettlement country (19–21). Specifically, refugee parents may value retaining their home culture (22), but refugee youth are often exposed to different cultural expectations when attending educational institutions (23, 24). This can result in conflicts between parents and children (19, 25). Overall, research has consistently shown that refugee youth are confronted with several stressors when adjusting to a new country which can negatively affect mental health (26–29).

The term “acculturation” has been used to describe the “meeting of cultures and the resulting changes” (30). Although all groups that come into contact may undergo change, in practice, changes are usually more pronounced in individuals who settle in a new country (and not vice versa). Berry (31) proposed a model for categorizing people’s acculturation orientations along two dimensions: (1) whether the heritage culture (also referred to as “minority” or “home” culture) is maintained after resettlement, and (2) whether the culture of the resettlement country (also referred to as “majority” or “host” culture) is adopted. Across these two dimensions, four distinct acculturation orientations emerge: Individuals who show an assimilation orientation adopt the culture of the resettlement country but do not maintain their heritage culture; those who show a separation orientation do not adopt the culture of the resettlement country but maintain their heritage culture; an integration orientation, also referred to as biculturalism, is shown by individuals who adopt the culture of the resettlement country while maintaining their heritage culture, and, finally, marginalization occurs when individuals neither maintain their heritage culture nor adopt the culture of the resettlement country. In the past years, a growing body of literature has examined the relationship between acculturation orientations and mental health in migrants and refugees. A systematic review and meta-analysis including 83 studies with immigrant minors and adults found that a bicultural orientation was associated with better psychosocial adjustment compared to a monocultural orientation (32). For immigrant youth, a (flexible) orientation toward both the heritage culture and culture of the resettlement country has also been connected to better psychological adjustment (33). Research with refugee youth from the Middle East showed that integration was associated with less internalizing symptoms, less post-migrations stressors and better sociocultural adjustment (34, 35).

However, the model proposed by Berry (31) has also received several critiques. It has been criticized that people’s acculturation orientations do not always fit into the four above mentioned categories and that the advantages or disadvantages of different orientations may depend on contextual and/or individual factors (24). For example, orientation toward the culture of the resettlement country has been associated with better mental health in educational and work settings, while orientation toward the heritage culture was preferable for mental health outcomes in private life (36). It has also been criticized that the model focuses predominantly on acculturation orientation of the immigrant or refugee groups while neglecting the role of the context in which these attitudes are shaped, for example state policies or acculturation attitudes of the majority population in the resettlement country (37). Furthermore, comparisons between studies can be difficult due to differences in methodologies and in how acculturation is operationalized (30).

In addition to measures of cultural orientation, other variables that are associated with refugee youth’ adjustment to life in their resettlement country may also play a role in refugee youth’s mental health. For instance, proficiency in the language of the resettlement country has been reported to be an important indicator of acculturation processes: Young refugees resettled in Australia who reported higher confidence in their language skills had fewer problems overall and settled in faster (38). Language skills have also been connected to better mental health in accompanied and unaccompanied refugee youth, for instance in Denmark, Germany, and Australia (9, 39, 40), and in Syrian refugee youth resettled in Germany (35). Another important indicator of acculturation is access to social networks. Specifically, social support has been consistently identified as a protective factor for mental health problems and PTSD (41, 42). For refugee youth, this includes support by family and relatives (43–45), but especially by peer networks and friends (43, 46–48). Other factors also play a role in refugee youths’ mental health outcomes, such as country of origin (11, 49), not having a secure status of residence in the resettlement country (49, 50), and a sense of belonging to their school (25, 51). These findings highlight the need to investigate a range of potential protective factors, in addition to cultural orientation, to gain a nuanced understanding of the relation between acculturative processes and mental health.

As outlined above, refugee youths belong to a vulnerable group. They may experience resettlement as particularly challenging due to the compounding effects of having to acculturate to a new country and facing the developmental tasks of adolescence (5). It is therefore important to identify potential protective factors that may foster mental health and adjustment after resettlement. This study focused on Arabic-speaking refugee youth in Germany. Since 2015 Europe, and more specifically Germany, has seen an unprecedented influx of refugees from the Middle East, predominantly by refugees from Syria and Iraq (52). At the time of the study, these two groups made up one third of the refugee population in Germany with most of them speaking Arabic as a mother tongue or as a second language (53). Given these number, it is essential to understand how Arabic-speaking refugee youth adjust to life in Germany, and which impact the transition has on their mental health and wellbeing. Our study had two aims. First, to test whether acculturation orientation is associated with depressive and posttraumatic stress symptoms. Second, to identify additional factors associated with acculturation processes that are associated with depressive and posttraumatic stress symptoms.

## 2. Materials and methods

### 2.1. Sample characteristics

We targeted Arabic-speaking refugee youth above the age of 14 who attended school in Berlin, Germany. Of the 112 students who agreed to participate, seven participants dropped out during data collection and were excluded from the study. Of the remaining 105 participants, two participants were excluded after data collection as they reported an age below 14, another two participants were excluded for the purpose of this analysis as they reported living unaccompanied in Germany and this study focused on youth living with their families. The final sample consisted of 101 refugee youths (52.3% females, none identified as “other”) living with their families, aged 14–20 years (*M* = 16.6, *SD* = 1.34). All participants spoke Arabic and came to Germany as refugees. Most participants originated from Syria (75.2%), followed by Iraq (10.9%), and Palestine (5.9%). Almost all participants (96.0%, *n* = 97) reported having experienced at least one potentially traumatic event with an average of nine potentially traumatic events per participant. When screened for symptoms of mental health problems, 12.9% (*n* = 13) of the total sample met the screening criteria for probable PTSD according to the PCL-5, and 57% (*n* = 58) scored above the cut-off for depression according to HSCL-25. See Table 1 for a detailed description of the sample.

**TABLE 1:** Sample characteristics.

	*n*	%	*M*	*SD*	Range
Female gender	53	52.5	–	–	
Age	–	–	16.6	1.34	14–20
Length of stay in Germany (in years)	–	–	3.12	1.32	1–7
Potentially traumatic experiences (PTE)	–	–	8.87	5.61	0–20
Secure asylum status	39	38.6	–	–	–
Country of origin					
Syria	76	75.2			
Iraq	11	10.9	–	–	–
Palestine	6	5.9			
Lebanon	3	3.0			
Egypt	1	1.0	–	–	–
Libya	1	1.0			
Bahrein	2	2.0			
Kuwait	1	1.0	–	–	–
Class: welcome-class	34	33.7	–	–	–
Friends					
All friends	–	–	7.95	8.18	0–30
Friends in Germany	–	–	5.73	6.31	0–30
Friends born in Germany	–	–	5.65	6.09	0–30
Depressive symptoms (HSCL-25)	–	–	1.95	0.55	1–3.40
Posttraumatic stress symptoms (PLC-5)	–	–	18.3	14.1	0–67
Acculturation Germany (unidimensional)	–	–	4.60	1.17	1.8–7
Acculturation heritage (unidimensional)	–	–	5.92	1.08	1.9–7

*N* = 101; HSCL-25: Hopkins-Symptom Checklist-25, PCL-5: PTSD-Checklist.

This study was part of a larger project investigating how newly arrived Arabic-speaking refugee youth adjust to life in Germany [for a qualitative study with a different sample of youth in the Berlin/Brandenburg area, see (4)]. To recruit participants, we contacted 418 schools in Berlin *via* email and informed them about the study. A total of 14 schools agreed to participate in the study. All other schools were either unavailable, had no students that fulfilled the inclusion criteria, withdrew their agreement to participate, or did not respond. Before the main study, we conducted a series of pilots to ensure that procedures and study materials were appropriate. First, we tested the full survey on a group of Arabic-speaking refugee adults as well as with one refugee adolescent. Thereafter, we piloted the survey with six Arabic-speaking refugee youths in one of the schools that had agreed to take part in the study. Participants’ feedback was included in the final survey and the six participants were excluded from the final data set.

Data collection took place in Berlin, Germany, between November 2018 and June 2019 and followed the same procedure at every school. For each school’s testing appointment, one native Arabic-speaking and one native German-speaking researcher were present. Arabic-speaking refugee youths aged 14 years and older were identified by the school and invited to take part in the study by the two researchers. In Berlin, most students arriving as refugees or immigrants start to attend so-called Willkommensklassen (welcome-classes) within 3 months of their arrival. These classes focus on language acquisition and on introducing newly arrived students to the German school system. As soon as students in welcome-classes are sufficiently fluent in German, they transfer to regular classes. For this study, students from both welcome-classes and regular classes were invited to participate. All contact was handled by the school.

Those who agreed to participate were gathered in a different classroom to ensure quiet surroundings. Every participant received a tablet on which the survey was presented in written form in both Arabic and German using the Software LimeSurvey (54). Before starting the survey, the students were informed about the details of the study and gave consent to participate (no parental consent was needed as adolescents were aged 14 years and older). A maximum of six students took part in each session, which lasted around 45 min on average. Both researchers were available to answer questions during the survey sessions and ensured that participants did not influence each other and answered the survey independently. For those students who were not able to read in Arabic due to interrupted education on the flight journey and had insufficient German language skills, questions were read quietly in Arabic by the Arabic-speaking researcher. At the end of the session, participants received a 12 Euro voucher from an electronics store and a list of mental health counseling services available in Arabic should the need for such services arise.

The Research Ethics Committee of the Department of Education and Psychology at Freie Universität Berlin the Berlin (203/2018) and the Berlin Senate’s Department for Education, Youth and Family approved this study.

### 2.2. Measures

For all measures, a three-step approach was used to obtain valid instruments as recommended for cross-cultural research (55). Firstly, measures were translated from English to German and Arabic. Secondly, another person translated the questionnaires back into English, and thirdly, differences between the two versions were discussed and the wording was revised accordingly. The scales concerning acculturation orientation and positive and negative inter- and intragroup contact were translated in the same way for a previous study (56).

#### 2.2.1. Sociodemographic variables

The survey included questions regarding participants’ age, gender (female, male, other), and country of origin. The socio-demographic survey also asked about participants’ flight to Germany (time of arrival), whether they lived alone or with their family, the type of class attended (welcome-class or regular class) and their asylum status. A confirmed asylum status was considered “secure,” while a temporary status or rejection were considered “insecure.”

#### 2.2.2. Traumatic exposure and posttraumatic stress symptoms

To assess exposure to potentially traumatic events, two standardized trauma lists, the Harvard Trauma Questionnaire [HTQ; (57)], focusing on war-related trauma and torture, and the Posttraumatic Diagnostic Scale (58, 59) focusing on civilian trauma, were combined. Specifically for the HTQ, the authors recommend modifying and adapting the questionnaire to the characteristics of each cultural group as traumatic events may vary depending on historical, political, and social context. Therefore, the combined trauma list was supplemented with two additional items, “ill-treatment by smugglers” and “violent attack by authorities” that were identified as frequently occurring on the Balkan route, one of the main flight routes for refugees from the Middle East in 2015/2016 (60). The resulting list was presented to the participants three times, asking for experiences in their home country, during their flight, and in Germany. For each list, events were rated as “experienced” “witnessed,” or “neither-nor.” For the current study, the three lists were combined, i.e., when a traumatic event was marked as “experienced” or “witnessed” in any of the three lists (in their home country, during their flight, in Germany) it was scored as 1, otherwise it was scored as 0, and a sum score across all trauma types was calculated.

Posttraumatic stress symptoms according to the DSM-5 criteria were assessed using the PTSD-Checklist for DSM-5 [PCL-5; (61)]. Participants answered 20 items on a five-point Likert scale (from 0 “not at all” to 4 “extremely”). Sum scores were calculated for the analysis. Reliability in the current study was excellent (Cronbach’s α = 0.92). To screen for a probable PTSD diagnosis, we followed the DSM-5 diagnostic rule. As recommended, each item rated as 2 = “Moderately” or higher was considered an endorsed symptom. Participants that met DSM-5 diagnostic criteria according to this recommendation were considered as probably having PTSD.

#### 2.2.3. Depressive symptoms

Depressive symptoms were assessed using the depression subscale of the Hopkins-Symptom Checklist-25 [HSCL-25; (62)]. The scale consists of 15 items that are scored on a four-point Likert scale (from 1 “not at all” to 4 “extremely”). Mean scores were calculated for the analysis. Values ≥ 1.75 indicate possible depression. Reliability in the current study was good (Cronbach’s α = 0.86).

#### 2.2.4. Acculturation related variables

Acculturation was assessed using several indicators to cover various facets of acculturation attitudes, behaviors, and competencies.

##### 2.2.4.1. Vancouver index of acculturation

The Vancouver index of acculturation (VIA) assesses orientation toward the heritage culture and the culture of the resettlement country (63). The 20 items were presented in pairs, with one item in each pair referring to the heritage culture and the other item referring to German culture, for example “I often participate in cultural traditions of the Syrian culture” or “I often participate in German cultural traditions.” Items were rated on a seven-point scale ranging from not at all (1) to very much so (7) with higher subscale scores indicating higher levels of orientation toward the culture represented. The VIA has been used in several studies with children and adolescents and proven a valid instrument (64–66). Recently, an Arabic translation of the VIA has been validated in 957 Syrian refugee children and youth aged 11–18 living in Turkey (67). Analyses confirmed the two-dimensional structure and showed good construct, convergent and discriminant validity as well as satisfying reliability coefficients. Reliability in the current study was excellent for the subscale concerning heritage culture (Cronbach’s α = 0.91) and good for the subscale concerning German culture (Cronbach’s α = 0.86).

##### 2.2.4.2. Positive and negative contact with heritage culture and German culture

To measure intergroup contact in Germany with persons from the heritage culture and the German culture, a short questionnaire was used that assesses quantity and quality of contact (68). For both cultural groups, eight items were presented to assess the amount of positive and negative contact, for example “How often do you have friendly contact with Germans?” or “How often did you have conflicts with people from your home country in Germany?” Items were rated on a 7-point scale ranging from very little (1) to very much (7). Reliability in the current study was acceptable for all four scales (Cronbach’s α = 0.71 to Cronbach’s α = 0.78).

##### 2.2.4.3. German language skills

Participants were asked to subjectively rate their German language skills on four dimensions: Speaking, writing, reading, and listening. Each scale ranged from 0 (“no ability”) to 4 (“very good”). For analyses, a mean score was calculated across all four dimensions with higher values indicating better German language skills.

##### 2.2.4.4. Friendship network

To assess participants friendship network, participants were asked three questions: (1) “How many close friends do you have?,” (2) “How many of your close friends live in Germany?,” and (3) “How many of your close friends were born in Germany?” For each question, the scale ranged from 0 to 30 friends.

### 2.3. Statistical analysis

To test for associations between acculturation orientations and mental health, all participants were assigned to one of the four acculturation orientations proposed by Berry (31). Following the procedure used by Behrens et al. (69), both scales of the VIA were split at the median to determine low vs. high orientation toward the heritage culture and the German culture, respectively. The four acculturation orientations were as follows: Separation (high heritage/low German orientation, *n* = 25), integration (high heritage/high German orientation, *n* = 24), marginalization (low heritage/low German orientation, *n* = 29), and assimilation (low heritage, high German orientation, *n* = 25). Afterward, Kruskal–Wallis tests were conducted to test for differences in depressive symptoms and posttraumatic stress symptoms between the four groups. To address critiques of the acculturation model proposed by Berry (31), we additionally performed Kruskal-Wallis tests for both scales separately (orientation toward heritage cultural group high/low and orientation toward German cultural group high/low).

To identify further indicators of acculturation, correlation analysis was conducted. Afterward, linear regression analysis was conducted separately for depressive and posttraumatic stress symptoms. All indicators that revealed significant correlations with either depressive or posttraumatic stress symptoms were included into the regression model. As male gender has been reported as a protective factor in several previous studies, gender was included as independent variable into the regression analysis, and gender-disaggregated regression analyses were additionally performed exploratory. For inclusion in the regression model, all categorical variables were dichotomized, and dummy coded with a reference category (70). For both regression models, Pagan–Breusch test indicated heteroskedasticity. Therefore, the Huber-White estimator of standard errors was applied for these regression analyses using the package “sandwich” (71, 72) in R. No further assumptions were violated. There were no missing data in the data set used for this analysis. All statistical analyses were conducted using R version 4.2.0 (73).

## 3. Results

### 3.1. Acculturation orientations and mental health

**FIGURE 1:**
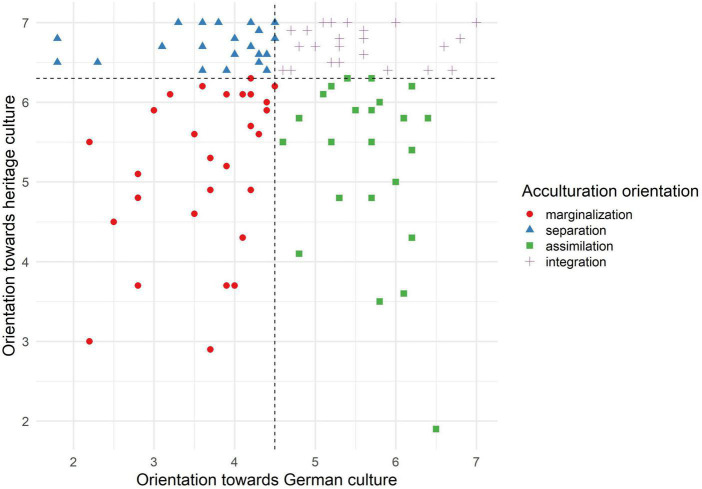
Scatterplot: displayed is the classification of the participants into the four acculturation orientations according to the definition by Berry (31): Separation (*n* = 25), integration (*n* = 24), marginalization (*n* = 29), and assimilation (*n* = 25).

Participants were grouped into one of the four acculturation orientations proposed by Berry (31) using both acculturation dimensions measured by the VIA. Figure 1 gives a detailed overview of the bivariate distribution and classification of the participants. On average, participants showed higher orientation toward their heritage culture (*Mdn* = 6.3) than toward the German culture (*Mdn* = 4.5). However, almost all participants reported bicultural orientation toward both cultural groups with high or very high orientation toward their heritage cultural group and medium to high orientation toward the German cultural group (see Figure 1).

**FIGURE 2:**
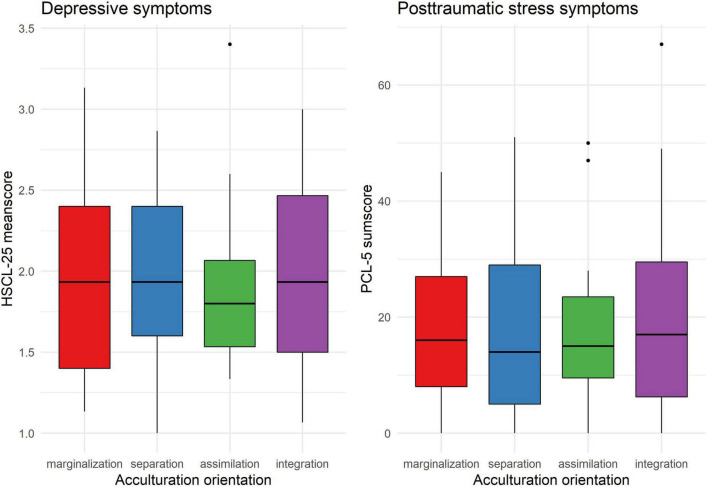
Mean scores of depressive symptoms and posttraumatic stress symptoms grouped by acculturation orientations.

In a second step, mean scores for depressive symptoms and posttraumatic stress symptoms were compared across the four acculturation orientations. Kruskal–Wallis rank sum tests revealed no significant differences for depressive symptoms or posttraumatic stress symptoms between the four groups. For details see Table 2 and Figure 2. As the model with four acculturation orientations proposed by Berry (31) has received several critiques, we additionally performed univariate analyses for cultural orientation toward the heritage culture (low vs. high) and to the German culture (low vs. high). Kruskal–Wallis rank sum tests revealed no significant differences for depressive symptoms or posttraumatic stress symptoms neither for cultural orientation toward the heritage culture nor for cultural orientation toward the German culture. The results of this additional analysis can be found in Supplementary material B.

**TABLE 2:** Mean scores of depressive symptoms and posttraumatic stress symptoms grouped by acculturation orientations.

Depressive symptoms				Kruskal–Wallis rank sum test		
	***n***	***M***	***SD***	**χ^2^**	***df***	***p***
Marginalization	29	1.95	0.57	0.519	3	0.915
Separation	25	1.96	0.54			
Assimilation	23	1.87	0.48			
Integration	24	1.99	0.61			
**Posttraumatic stress symptoms**				**Kruskal–Wallis rank sum test**		
	***n***	***M***	***SD***	**χ^2^**	***df***	***p***
Marginalization	29	18.8	13.1	0.263	3	0.967
Separation	25	16.7	14.4			
Assimilation	23	17.5	12.5			
Integration	24	20.3	16.8			

Posttraumatic stress symptoms were measured with the PTSD symptoms Checklist for DSM-5 (PCL-5); depressive symptoms were measured with the Hopkins-Symptom Checklist-25 (HSCL-25).

### 3.2. Additional indicators of acculturation as covariates of mental health in refugee youth

Several additional indicators of acculturation were assessed as potential covariates of mental health in refugee youth. As expected, correlations between depressive and posttraumatic stress symptoms were high (*r* = 0.63). Significant correlations were found for several indicators of acculturation with both depressive and posttraumatic stress symptoms. Depressive symptoms correlated negatively with German language skills and the number of close friends in Germany. Posttraumatic stress symptoms correlated positively with age, and the amount of traumatic exposure and negatively with the number of close friends in Germany. All correlation coefficients with mental health outcomes are reported in detail in Table 3. A comprehensive correlation matrix of all variables can be found in Supplementary material A.

**TABLE 3:** Correlations of indicators of acculturation with depressive symptoms and posttraumatic stress symptoms.

	Depressive symptoms	Posttraumatic stress symptoms
Age	0.08	0.25*
Female gender (vs. male gender)^a^	0.04	−0.01
Length of stay in Germany (in years)	0.00	0.08
Home country Syria (vs. all other countries)^b^	0.13	0.09
Secure asylum status (vs. insecure asylum status)^c^	−0.11	0.01
Welcome class (vs. regular class)^d^	−0.04	−0.05
German language skills	−0.22*	−0.14
Potentially traumatic events, aggregated	0.09	0.38***
Acculturation Germany (unidimensional)	0.00	0.00
Acculturation heritage (unidimensional)	−0.01	0.00
Positive contact Germans	−0.09	0.08
Negative contact Germans	0.02	0.03
Positive contact with people of same heritage	−0.07	0.08
Negative contact with people of same heritage	0.02	0.04
Friends in Germany	−0.26**	−0.26**
All friends	−0.09	−0.10
Friends born in Germany	−0.10	−0.03

*N* = 101. Posttraumatic stress symptoms were measured with the PTSD symptoms Checklist for DSM-5 (PCL-5); depressive symptoms were measured with the Hopkins-Symptom Checklist-25 (HSCL-25). Acculturative orientations were measured with the Vancouver acculturation index (VIA).

^a^0: Male; 1: Female.

^b^0: Country of origin: All other countries; 1: Country of origin: Syria.

^c^0: Insecure asylum status; 1: Secure asylum status.

^d^0: Attending regular class; 1: Attending welcome class (preparatory class with only refugee/immigrant youth focusing on language acquisition).

*p < 0.05, **p < 0.01, ***p < 0.001.

Age, potentially traumatic events, language skills, and friends in Germany showed significant associations with either depressive or posttraumatic stress symptoms and were therefore included as predictors in the regression analysis. Additionally, gender and length of stay in Germany were included as they are established risk factors for mental health in refugee youth (74). Results of the regression analyses are reported in Tables 4, 5. For depressive symptoms, better language skills and a higher number of friends in Germany were significantly related with lower symptom scores. For posttraumatic stress symptoms, a higher number of friends in Germany was significantly related with lower symptom scores. Additionally, participants who had experienced a higher number of traumatic events showed higher posttraumatic stress symptom scores.

**TABLE 4:** Regression analysis for depressive symptoms.

Depressive symptoms				
	***B***	***SE***	**β**	***p***
Intercept	2.37	0.76		0.002**
Female gender^a^	0.00	0.11	0.00	0.980
Age	0.01	0.04	0.02	0.866
Length of stay in Germany (in years)	0.02	0.04	0.05	0.652
Potentially traumatic events	0.01	0.01	0.06	0.544
Language skills	−0.18	0.07	−0.21	0.016*
Friends in Germany	−0.02	0.01	−0.25	0.006**

*R*^2^ = 0.12; *R*^2^*_adj_* = 0.07; depressive symptoms were measured with the Hopkins-Symptom Checklist-25 (HSCL-25).

^a^0: Male; 1: Female.

*p < 0.05, **p < 0.01.

**TABLE 5:** Regression analysis for posttraumatic stress symptoms.

Posttraumatic stress symptoms				
	***B***	***SE***	**β**	***p***
Intercept	4.43	18.40		0.810
Female gender^a^	−0.23	2.85	0.00	0.936
Age	1.48	1.00	0.14	0.140
Length of stay in Germany (in years)	0.51	0.98	0.05	0.605
Potentially traumatic events	0.84	0.28	0.33	0.003**
Language skills	−2.57	1.85	−0.12	0.167
Friends in Germany	−0.54	0.17	−0.23	0.002**

*R*^2^ = 0.25; *R*^2^*_adj_* = 0.20; posttraumatic stress symptoms were measured with the PTSD symptoms Checklist for DSM-5 (PCL-5).

^a^0: Male; 1: Female.

**p < 0.01.

Exploratory regression analysis performed separately for male and female participants revealed that associations were more pronounced in female participants than in males. For female participants, the results of the aggregated analysis were confirmed while analyses for male participants showed no associations between mental health symptoms and friendship networks or language skills, respectively. The amount of explained variance was higher in the sub-group analysis for female participants than for male participants for both depressive symptoms (*R*^2^_*adj*_ = 0.25 vs. *R*^2^_*adj*_ = 0.08) and posttraumatic-stress symptoms (*R*^2^_*adj*_ = 0.33 vs. *R*^2^_*adj*_ = 0.25). The results are reported in detail in Supplementary materials C, D.

## 4. Discussion

This study investigated the relationship between acculturation and mental health in Arabic-speaking refugee youth living with their parents in Berlin, Germany. In a first step, we analyzed associations between the four acculturation orientations proposed by Berry (31) and depressive and posttraumatic stress symptoms. In a second step, we explored whether several other indicators of acculturation were related with depressive and posttraumatic stress symptoms.

We found that acculturation orientation was not associated with depressive or posttraumatic stress symptoms in our sample. This contrasts with previous findings that have shown better mental health outcomes for immigrants and refugees with bicultural orientation who maintain some contact with their home culture and, at the same time, attempt to connect with the culture of the resettlement country (32, 74). Also, bivariate analyses revealed no associations between acculturation scales and mental health outcomes, neither for the unidimensional scales of cultural orientation toward heritage culture and German culture nor for scales measuring positive and negative contact with both cultures.

A possible explanation for these results may be that our sample differed from other studies in the field regarding several characteristics. First, our study focused on Arabic-speaking refugee youth who lived with their families in Berlin, Germany, and attended school on a regular basis at the time of the study. To date, most research on acculturation and mental health has focused on immigrants or unaccompanied refugee minors (32, 33, 44, 69, 75). Pathways of stressors and resilience may differ for these groups as every group faces specific challenges that are unique to their living situation (9, 39). It is possible that integration is a more favorable strategy for adults and unaccompanied refugee youth as they are under more pressure to function in the new society on their own. In contrast, for accompanied refugee youth, orientation toward the new culture may also lead to increased conflicts with their parents due to potential differences in cultural values (19, 20, 23). Associations between acculturation orientation and mental health may be highly dependent on contextual factors and family dynamics (24) and may vary across life domains. While orientation toward the majority culture in work or school contexts has been related to better mental health, in private life orientation toward heritage culture has been related to better mental health (36). Consequently, there may be no “best way” to cope with the challenges of acculturation for this group. This might explain, why in our sample, none of the acculturation styles was associated with mental health outcomes.

Moreover, other characteristics of our sample and contextual factors may have contributed to the results. While the sample is comparable to other studies concerning the prevalence of mental health problems (2, 9), it may be unique due to several reasons: The study was conducted in Germany’s capital Berlin which is multicultural and metropolitan, and where particularly high numbers of refugees from Syria have arrived since 2015 due to the ongoing war. Most participants in our study came from Syria and, at the time the study took place in 2018/2019, they had already stayed in Germany on average for 3 years. In addition, more than half of the participants were female. Although this may not be representative for refugee youth populations, it nevertheless reflects typical sample characteristics that have been found in Syrian refugees arriving in Europe at the time (52). However, other studies investigating acculturation orientations and mental health in Middle-Eastern refugee youth mainly included male participants (35) or exclusively focused on male refugee youth (34), which may also explain why our results diverged from previously published studies.

Furthermore, several methodological aspects must be considered. First, our sample showed high levels of orientation toward both cultures but especially high orientation toward the heritage culture. As a result, variability on these scales was low and may have contributed to the non-significant results. For analyses, we assigned all participants to one of the four acculturation orientations using a median split, thus the grouping into one of the four acculturation orientations was dependent on the distribution of ratings in the sample and relative to the studied group. Few participants in our study showed low orientation to either cultural group in terms of absolute numbers, and almost all participants, to some extent, showed bicultural orientation. Some researchers have therefore criticized the median-split approach as arbitrary and have suggested using person-centered approaches such as latent profile analysis to ensure the validity of the categorization (76, 77). Unfortunately, due to the limited sample size this was not possible in the current study. However, considering the critique that the categorical model proposed by Berry (31) has received (78), we have additionally performed analyses with the unidimensional scales of cultural orientation. These analyses, too, did not reveal significant associations between acculturation orientation and mental health. Therefore, we consider our results as relatively robust concerning the chosen method of analysis. Finally, it must be noted that the VIA assesses cultural orientation. Thereby, it is a measure of attitudes and does not directly refer to actual experiences, behavior, or skills which may show stronger associations with mental health symptoms.

In addition to acculturation orientations, we also investigated whether other indicators of acculturation may act as protective factors for mental health in young refugees. We found that the number of friends in Germany was negatively associated with both depressive and posttraumatic stress symptoms. Social support is a well-established protective factor in trauma survivors (41, 42) and previous research has stressed the importance of friendships for refugee youth (43, 46). Some studies have suggested that ethnicity of peers and social networks may influence the effects of social support on mental health in refugee children and youth (40, 79). However, in our study, the number of close friends originating from Germany, in contrast to the general number of friends in Germany, was not significantly associated with mental health symptoms. A recent study with refugee youth in Belgium found that peers of similar heritage were more important in the early stages of flight, while local friends in the resettlement country became increasingly important in later stages of the resettlement process (43). Overall, this suggests that the proximity of friends in everyday life—but not ethnicity—mattered most for youth in our sample, most of whom had been in Germany for a while. Access to joining local clubs or sport teams may provide refugee youth with vital opportunities for building local peer networks (38, 80).

Furthermore, we found that better German language skills were significantly associated with lower symptoms of depression. This is in line with studies showing associations between language skills and symptoms of depression in refugee minors (9, 40). Language has been found to be a major contributor for successfully dealing with resettlement, and language skills are typically fostered to allow refugee youth to attend school. Our findings suggest that language skills may not only be associated with educational success but also significantly linked to mental health. Fostering language acquisition in refugee youth may thus not only support their academic development, but also have a positive effect on their mental health. In Berlin, most students arriving as refugees attend so-called welcome-classes that focus primarily on language acquisition and may provide refugee children with a “safe space” (81). However, welcome-classes have also been criticized for separating refugee minors from students attending regular classes (82, 83), which may result in lower participation in school based extracurricular activities among young refugees (82). This, in turn, may lead to less opportunities for building local peer-networks and making friends—the second factor that was associated with lower mental health symptoms in our study. Policies should make sure that these two potentially protective factors are not mutually exclusive in practice and that refugee youth can acquire language skills and make friends at the same time.

Finally, to identify potentially gender-dependent associations, exploratory regression analysis was performed separately for male and female participants. Results indicated that associations were more pronounced in female participants than in male participants and that the model fit was better in the female sub-sample than in the male sub-sample. While these results must be interpreted with caution due to the small sample size, they support studies that have highlighted the importance of gender aspects in acculturation research and should be considered in future studies (84, 85).

### 4.1. Strengths and limitations

Few studies to date have investigated the association between acculturation and mental health in refugee youth living with their families. By focusing on acculturation orientations and other indicators of acculturation, we provide a nuanced picture of potential protective factors for refugee mental health in a high-income country (Germany). However, our study has several limitations. First, relatively few schools in Berlin agreed to participate in the study and our sample may not be representative of the refugee youth population at the time in Berlin. Second, all analyses were conducted based on cross-sectional data and, therefore, no causal inferences are possible. Longitudinal studies are needed for a better understanding of acculturation processes in the context of developmental pathways. Third, the number of friends and language skills were self-assessed by the students. While this approach most directly reflects how students see themselves and was therefore considered to be an adequate representation of social integration, it also bears the risk of over- or underestimation. Fourth, several factors limit the generalizability of our results. Socioeconomic status and post-migration experiences were not assessed in the study and could not be controlled for in the analyses. Moreover, our sample included a set of heterogenous countries with most participants originating from Syria, therefore especially the result concerning country of origin should be interpreted with caution. Finally, due to the highly skewed distributions on the scales measuring cultural orientation and the chosen method for analyzing associations between cultural orientation and mental health, conclusions outside the studied group must be drawn with caution.

### 4.2. Conclusion and implications for practice

Adjusting to a new culture after forced resettlement can be a stressful process for Arabic-speaking refugee youth in high income resettlement countries and negatively impact their mental health. While we found no significant relation between refugee youths’ acculturation orientations and depressive and posttraumatic stress symptoms, two other acculturative factors were significantly associated with mental health: better self-assessed German language skills and a higher number of friends in Germany. Previous research proposed several policy implications for fostering mental wellbeing of refugee youth in resettlement countries. These include rapid resolution of asylum claims, protection from post-migration violence, prioritizing family reunions, and providing physical and psychological healthcare (74). These measures are important and should be enforced with the most possible urgency. However, implementation depends to a large extent on the political will and changes may require resources and time. While there is no adequate substitute for these much-needed policy changes, the present study suggests that to prevent mental health problems in refugee youth, additional and relatively easy steps such as providing access to high-quality language classes and social activities with peers may have a positive impact on mental health.

## Data availability statement

The datasets presented in this article are not readily available because they contain information that could compromise the privacy of research participants. Requests to access the datasets should be directed to caroline.meyer@fu-berlin.de.

## Ethics statement

The studies involving human participants were reviewed and approved by the Research Ethics Committee of the Department of Education and Psychology at Freie Universität Berlin the Berlin (203/2018). Written informed consent from the participants’ legal guardian/next of kin was not required to participate in this study in accordance with the national legislation and the institutional requirements.

## Author contributions

LA, CK, PK, NS, FS, JSW, and RK conceptualized the study. LA coordinated the data collection. PK supervised the data collection and conceptualized of the study. CM performed the data analyses and drafted the manuscript. LA, NS, CK, PK, FS, JSW, and RK provided critical revisions to the manuscript. All authors read and approved the final manuscript.
