# Supplementary material for: With a little help from my friends? Acculturation and mental health in Arabic-speaking refugee youth living with their families
Source: Front Psychiatry. 2023 Mar 15;14:1130199. doi: 10.3389/fpsyt.2023.1130199 (PMC10061544; doi:10.3389/fpsyt.2023.1130199)
Supplement: Supplementary file 1 [file Data_Sheet_1.pdf]

Appendix A: Correlation matrix

	1	2	3	4	5	6	7	8	9	10	11	12	13	14	15	16	17	18	19
1. Depressive symptoms																			
2. Posttraumatic stress symptoms	0.63***																		
3. Age	0.08	0.25*																	
4. Female gender (vs. male gender) [a]	0.04	-0.01	0.00																
5. Length of stay in Germany (in years)	0.00	0.08	0.21*	-0.02															
6. Flight journey by land (vs. journey by plane) [b]	-0.04	0.07	0.14	-0.13	0.40***														
7. Home country Syria (vs. all other countries) [c]	0.13	0.09	0.05	0.28**	-0.04	-0.21*													
8. Secure asylum status (vs. insecure asylum status) [d]	-0.11	0.01	0.08	-0.06	0.28**	0.29**	0.03												
9. Welcome class (vs. regular class) [e]	-0.04	-0.05	-0.24*	0.01	-0.69***	-0.22*	-0.03	-0.05											
10. German language skills	-0.22*	-0.14	-0.09	-0.02	0.10	-0.14	0.00	0.03	-0.19										
11. Potentially traumatic events, aggregated	0.09	0.38***	0.20*	-0.19	0.15	0.16	-0.07	-0.08	-0.12	-0.02									
12. Acculturation Germany (unidimensional)	0.00	0.00	-0.06	-0.27**	-0.04	0.05	0.11	0.05	0.14	0.01	0.19								
13. Acculturation heritage (unidimensional)	-0.01	0.00	0.09	0.15	-0.09	-0.07	0.11	0.02	0.16	0.04	-0.05	0.08							
14. Positive contact Germans	-0.09	0.08	-0.04	-0.15	0.16	0.11	0.14	0.01	-0.23*	0.15	0.13	0.42***	-0.01						
15. Negative contact Germans	0.02	0.03	0.05	-0.10	0.31**	0.05	0.19	-0.02	-0.31**	0.05	0.13	0.04	-0.01	0.06					
16. Positive contact with people of same heritage	-0.07	0.08	-0.03	-0.08	-0.07	0.01	-0.05	-0.01	-0.03	0.03	0.09	0.15	0.49***	0.31**	0.02				
16. Negative contact with people of same heritage	0.02	0.04	0.12	-0.39***	0.17	0.24*	-0.18	0.11	-0.13	-0.05	-0.06	0.06	-0.14	0.06	0.37***	0.07			
18. Friends in Germany	-0.26**	-0.26**	-0.09	-0.19	0.14	0.04	-0.04	0.11	0.06	0.03	-0.06	0.11	0.09	0.17	0.05	0.14	0.03		
19. All friends	-0.09	-0.10	-0.02	-0.15	0.11	-0.06	-0.09	0.02	-0.02	0.03	0.06	0.16	0.05	0.23*	-0.12	0.13	0.08	0.62***	
20. Friends born in Germany	-0.10	-0.03	-0.08	-0.13	0.16	0.05	-0.08	0.14	-0.06	0.22*	-0.06	0.18	0.19	0.14	0.11	0.11	0.17	0.31**	0.22*

Note. $N = 101$. Posttraumatic stress symptoms were measured with the PTSD symptoms Checklist for DSM-5 (PCL-5); depressive symptoms were measured with the Hopkins-Symptom Checklist-25 (HSCL-25). Acculturative orientations were measured with the Vancouver Acculturation Index (VAI). [a]0: Male; 1: Female. [b]0: Flight by plane; 1: Flight by land. [c]0: Country of origin: All other countries; 1: Country of origin: Syria. [d]0: Insecure asylum status; 1: Secure asylum status. [e]0: Attending regular class; 1: Attending welcome class (preparatory class with only refugee/immigrant youth focusing on language acquisition). $^{*}p < .05$. $^{**}p < .01$. $^{***}p < .001$.

Appendix B: Unidimensional Analysis of Acculturation Orientation

Mean scores of depressive symptoms and posttraumatic stress symptoms grouped by acculturative orientation towards heritage culture (low vs. high) and German culture (low vs. high)

**Depressive symptoms**				Kruskal-Wallis rank sum test		
	n	*M*	*SD*	χ^2	*df*	*p*
**Orientation towards heritage culture**						
low	52	1.97	0.6	0.400	1	.527
high	49	1.92	0.5			
**Orientation towards German culture**						
low	51	1.94	0.5	0.098	1	.755
high	50	1.96	0.6			

**Posttraumatic stress symptoms**				Kruskal-Wallis rank sum test		
	n	*M*	*SD*	χ^2	*df*	*p*
**Orientation towards heritage culture**						
low	52	18.4	15.2	0.03	1	.862
high	49	18.2	13.0			
**Orientation towards German culture**						
low	51	18.7	14.8	0.03	1	.870
high	50	18.0	13.6			

Note. Posttraumatic stress symptoms were measured with the PTSD symptoms Checklist for DSM-5 (PCL-5); depressive symptoms were measured with the Hopkins-Symptom Checklist-25 (HSCL-25).

Mean scores of depressive symptoms and posttraumatic stress symptoms grouped by acculturative orientation towards the heritage culture (high vs. low) and the German culture (high vs. low).

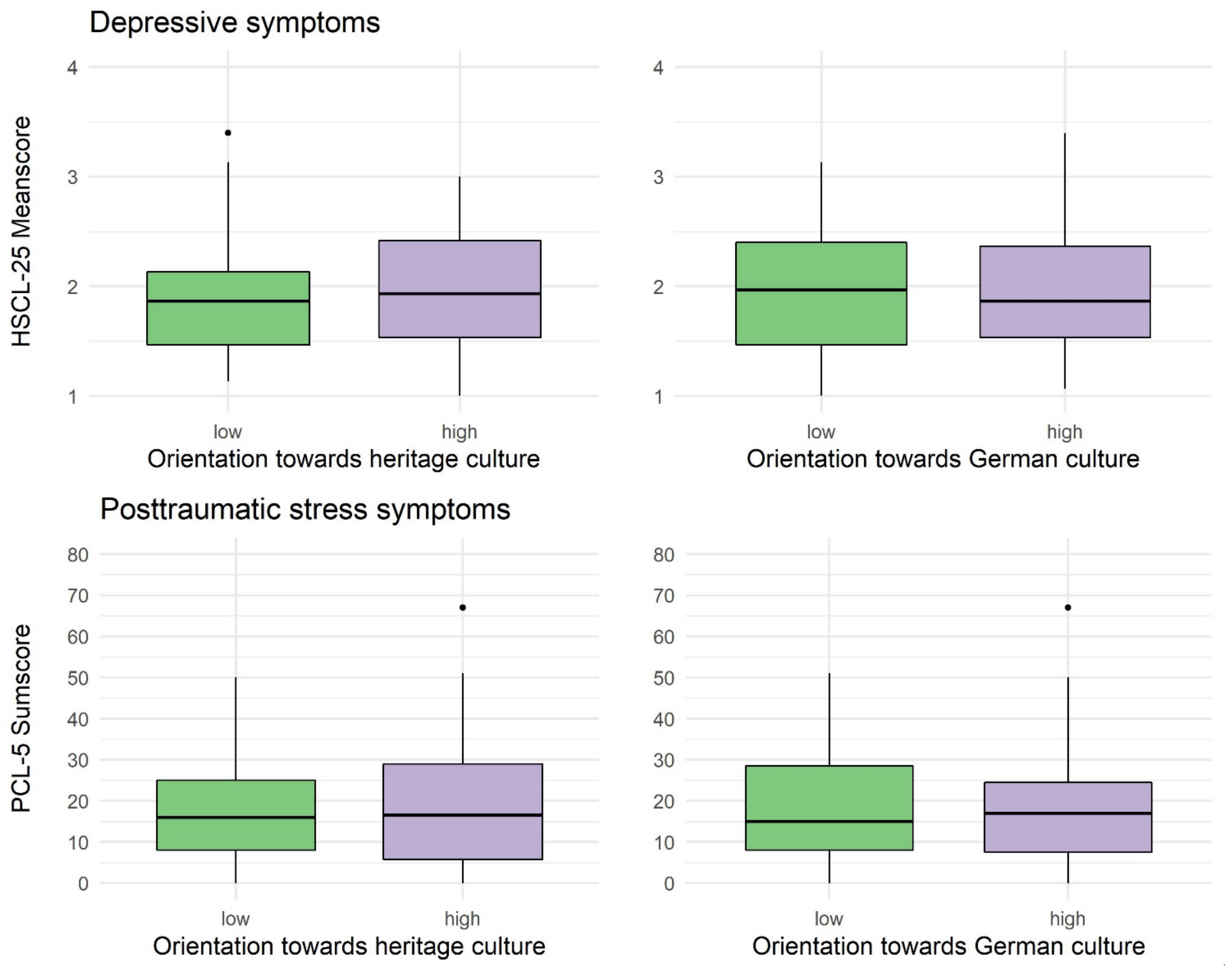

Appendix C: Gender-disaggregated regression analyses for female participants

Regression analysis for depressive symptoms and posttraumatic stress symptoms for female participants.

Depressive symptoms			
	B	*SE*	*p*
Intercept	3.16	0.92	.001**
Age	-0.03	0.05	.535
Length of stay in Germany (in years)	0.08	0.05	.127
Potentially traumatic events	0.00	0.01	.699
Language skills	-0.28	0.09	.004**
Friends in Germany	-0.03	0.01	.020*

Note. $R^2 = .25$; adj. $R^2 = .16$; depressive symptoms were measured with the Hopkins-Symptom Checklist-25 (HSCL-25).
$^{*}p < 0.05$, $^{**}p < 0.01$.

Posttraumatic stress symptoms			
	B	*SE*	*p*
Intercept	-41.46	27.26	.401
Age	3.89	1.66	.973
Length of stay in Germany (in years)	-0.89	1.49	.269
Potentially traumatic events	0.45	0.37	.001**
Language skills	-1.18	3.61	.095
Friends in Germany	-0.41	0.29	.039*

Note. $R^2 = .33$; R^2 Adj. = .26; posttraumatic stress symptoms were measured with the PTSD symptoms Checklist for DSM-5 (PCL-5).
$^{*}p < 0.05$, $^{**}p < 0.01$.

Appendix D: Gender-disaggregated regression analyses for male participants

Regression analysis for depressive symptoms and posttraumatic stress symptoms for male participants.

Depressive symptoms			
	B	*SE*	*p*
Intercept	1.32	1.19	.272
Age	0.05	0.07	.466
Length of stay in Germany (in years)	-0.05	0.07	.447
Potentially traumatic events	0.00	0.02	.881
Language skills	-0.01	0.16	.931
Friends in Germany	-0.02	0.01	.177

Note. $R^2 = .08$; adj. $R^2 = -.02$; depressive symptoms were measured with the Hopkins-Symptom Checklist-25 (HSCL-25).

Posttraumatic stress symptoms			
	B	*SE*	*p*
Intercept	-41.46	27.26	.136
Age	3.89	1.66	$.024^{*}$
Length of stay in Germany (in years)	-0.89	1.49	.554
Potentially traumatic events	0.45	0.37	.229
Language skills	-1.18	3.61	.745
Friends in Germany	-0.41	0.29	.167

Note. $R^2 = .25$; R^2 Adj. = .16; posttraumatic stress symptoms were measured with the PTSD symptoms Checklist for DSM-5 (PCL-5).
$^{*}p < 0.05$.
